# Medication non-adherence and its associated factors among heart failure patients in Africa: A systematic review and meta-analysis

**DOI:** 10.1016/j.ghrp.2026.06.007

**Published:** 2026-06-26

**Authors:** Tesfaye Birhanu Abebe, Teklestadik Teklesillasie Alemayehu, Tadele Mesfin Demelash, Hailemaryam Yohannes Asefa, Zena Admasu Yferu, Leul Wondemu Sebsebe, Redeat Dereje Tilahun, Wasihun Nigdu Mengestu, Melake Getahun Fikru, Nardos Teshome Gurmu, Abera Girma Beyena, Gebremariam Wulie Geremew, Yohana Moges Mehari, Temesgen Getachew Shiferaw, Hana Girma Abera, Diro Ajema Cheka, Bezawit Dereje Tilahun, Yilkal Abebaw Wassie

**Affiliations:** a School of Medicine, Collage of Health Science, Salale University, Fitche, Ethiopia; b School of Pharmacy, College of Medicine and Health Science, University of Gondar, Gondar, Ethiopia; c School of Medicine, Asrat Woldeyes Health Science Campus, Debre Berhan University, Debre Berhan, Ethiopia; d School of Medicine, College of Medicine and Health Science, University of Gondar, Gondar, Ethiopia; e School of medicine, Asella Referral and Teaching Hospital, Arsi University, Arsi, Ethiopia; f School of medicine, College of Health Science, Mekelle University, Mekelle, Ethiopia; g Department of Internal Medicine, St. Paul’s Hospital Millennium Medical College, Addis Ababa, Ethiopia; h Department of Medical Nursing, School of Nursing, College of Medicine and Health Sciences, University of Gondar, Gondar, Ethiopia

**Keywords:** Heart failure, Medication non-adherence, Africa, Systematic review, Meta-analysis

## Abstract

**Background:**

Medication non-adherence is a critical determinant of poor outcomes in heart failure, a condition posing a significant public health burden across Africa. Unique regional challenges such as healthcare resource constraints, economic disparities, and diverse healthcare systems exacerbate this issue. However, a consolidated estimate of non-adherence prevalence and its associated factors across the continent remains unknown.

**Methods:**

This systematic review and meta-analysis followed PRISMA guidelines (PROSPERO: CRD420251139486). A comprehensive search of databases (PubMed/MEDLINE, African Journal Online, and Google Scholar) was conducted for studies published between 2000 and 2025. Observational studies reporting non-adherence prevalence or associated factors among African heart failure patients were included. Study quality was assessed using the Joanna Briggs Institute (JBI) critical appraisal checklists for cross-sectional and cohort studies. Data were extracted and pooled using a random-effects model in STATA 17.

**Results:**

Seventeen studies (n = 6276 patients) from multiple African regions were included. The pooled prevalence of medication non-adherence among heart failure patients in Africa was 41.88% (95% CI: 25.43–58.34). Extreme heterogeneity (I² = 99.7%) was explored via subgroup analyses, revealing significant regional variation with Western Africa showing the highest prevalence (77.59%). Meta-analysis of associated factors identified lack of health insurance as the strongest predictor (OR = 4.27, 95% CI: 1.62–11.26). Other significant factors included polypharmacy (OR = 2.58, 95% CI: 2.00–3.33) and presence of comorbidities (OR = 1.76, 95% CI: 1.31–2.35). No publication bias was detected (Egger's test: *p* = 0.267).

**Conclusions:**

Approximately two in five heart failure patients in Africa are non-adherent to their medication, with prevalence rates varying significantly by region. This rate is exacerbated by financial, clinical, and therapy-related factors. Concerted multi-faceted interventions are urgently needed across the continent. Recommendations include expanding health insurance coverage, implementing medication subsidy programs, strengthening therapy simplification strategies, and developing context-specific adherence support strategies tailored to regional healthcare contexts and patient populations.

## Introduction

Heart failure presents a critical public health challenge across Africa, where structural healthcare constraints and socioeconomic factors converge to create complex barriers to effective disease management.^1^ The continent bears a disproportionate heart failure burden characterized by early disease onset, high mortality rates, and unique etiological patterns that distinguish it from high-income regions.^2^ Within this context, medication adherence emerges as a pivotal determinant of clinical outcomes, yet remains substantially compromised by multifaceted challenges.^3^

The World Health Organization's framework conceptualizes medication adherence through five interdependent dimensions that are particularly relevant to the African context.^4^, ^5^ Socioeconomic factors encompass financial barriers, health insurance coverage limitations, and transportation challenges that frequently prevent patients from obtaining and maintaining prescribed therapies.^6^ Healthcare system-related factors include medication availability inconsistencies, fragmented care coordination, limited follow-up mechanisms, and workforce shortages that undermine continuous care delivery.^7^ Condition-related factors involve the asymptomatic nature of compensated heart failure, high comorbidity burden, and mental health considerations that affect self-management behaviors.^8^ Therapy-related factors comprise complex dosing regimens, side effect profiles, and medication costs that create practical barriers to adherence.^9^, ^10^ Patient-related factors incorporate health literacy levels, cultural beliefs about illness and treatment, and social support systems that influence medication-taking behaviors.^11^ The interplay of these five dimensions creates substantial challenges for medication adherence across African healthcare settings. Financial constraints remain particularly salient, with out-of-pocket payments for medications and transportation costs frequently exceeding household resources. Healthcare system limitations, including stockouts of essential cardiovascular medications and limited access to follow-up care, further compromise adherence.^12^ Additionally, low health literacy levels and cultural beliefs about chronic disease management create significant patient-related barriers. The clinical consequences of non-adherence are severe in the African context, contributing to increased hospitalization rates, accelerated disease progression, and premature mortality.^13^, ^14^, ^15^ These adverse outcomes not only affect individual patients but also strain already overburdened healthcare systems through cyclical patterns of decompensation and readmission. Beyond the clinical impact, addressing medication non-adherence carries substantial implications for health systems and policy. Identifying the pooled prevalence and key determinants of non-adherence would enable healthcare providers to target high-risk patients and design individualized support interventions, while region-specific estimates could guide geographically targeted public health programs. For policymakers, evidence on the financial and structural barriers to adherence—such as lack of health insurance and medication affordability—could inform decisions on expanding coverage, subsidizing medications, and strengthening primary care follow-up systems.^16^ For future research, this synthesis highlights critical evidence gaps, particularly in understudied regions, and guides the development of standardized adherence measurement tools validated for African populations.

This systematic review and meta-analysis therefore aims to synthesize available evidence through the WHO's multidimensional framework to establish consolidated prevalence estimates and quantitatively assess the strength of association between key determinants across all five dimensions. The findings will provide a comprehensive evidence base to inform targeted interventions and policies aimed at improving medication adherence and clinical outcomes in African heart failure populations.

## Methods

This systematic review and meta-analysis was conducted in accordance with the Cochrane Handbook for Systematic Reviews of Interventions and reported following the PRISMA guidelines.^17^ This meta-analysis was performed to determine the pooled prevalence and predictors of medication non-adherence among heart failure patients in Africa. The investigation was framed using a PICO framework, focusing on this patient population (P) and the outcomes (O) of adherence prevalence and its determinants. The review protocol was registered with PROSPERO (CRD420251139486).

### Search strategy

We searched electronic databases for all English-language and French-language articles published from inception until 29 August 2025. A comprehensive search strategy was carried out, employing digital databases including PubMed/MEDLINE, AJOL (African Journals Online), and Google Scholar. Searching terms for the techniques were “heart failure,” “medication adherence,” “treatment compliance,” “associated factors,” “predictor factors,” and “Africa,” combined with the names of all African countries. The initial search strategy was adapted in the MEDLINE database by blending Medical Subject Headings (MeSH) terms, text words, and BOOLEAN operators (OR, AND). It was then adjusted for use in various databases between 19 June 2025 and 29 August 2025. To supplement the primary database searches and minimize publication bias, we actively attempted to gather further information through multiple approaches. Subject matter experts were consulted, grey literature sources including conference proceedings were reviewed, manual reference list checking (snowballing) of all included studies was independently performed by two reviewers, and authors of primary studies were contacted directly for missing data. However, despite these thorough efforts, no additional studies meeting our inclusion criteria were identified beyond those captured through the primary database searches.

### Eligibility criteria: inclusion criteria and exclusion criteria

This review included primary research studies involving individuals of any age diagnosed with heart failure in African countries. Eligible publications comprised peer-reviewed original articles, academic theses, and scientific conference abstracts published in English or French between 2000 and 2025. Studies were required to explicitly report both sample size and research setting. We included observational studies—specifically cross-sectional, cohort, or case-control designs—where the primary outcome was medication non-adherence. Eligible studies reported either the prevalence of medication non-adherence or examined factors associated with non-adherence using multivariate analysis. All included studies focused on heart failure patients receiving pharmacological therapy within African healthcare settings.

The following were excluded:

• Interventional studies, systematic reviews, meta-analyses, case reports, and editorials
• Studies lacking heart failure-specific data
• Publications with insufficient methodological detail, even after contacting authors
• Duplicate publications with overlapping datasets
• Studies addressing broader cardiovascular conditions unless heart failure patients comprised more than 50% of the sample
• Studies reporting only adherence levels without analysis of associated factors
• Research primarily focused on patients with multimorbidity

Additionally, we excluded pilot studies, qualitative investigations, validation studies, unpublished data, and seminar presentations. Studies with incomplete data that precluded meta-analysis, despite attempts to contact authors, were also excluded. Any uncertainties regarding eligibility were resolved through consensus among three independent reviewers (TMD, GWG, and BDT).

### Study selection procedures

To manage the search results, all records were imported into EndNote X9, where duplicates were identified and removed. The screening was conducted in two phases by independent reviewers (TBA, TTA). The initial phase involved title/abstract screening, followed by a comprehensive full-text assessment of potentially eligible studies. Disagreements at any stage were adjudicated by a group of additional researchers (YAW, YDS, LWS, DAC, and RDT). The PRISMA flow diagram outlines the complete selection process from identification to final inclusion.

### Quality assessment

Study quality was assessed using the Joanna Briggs Institute (JBI) critical appraisal tools appropriate to each study design. For the cross-sectional studies, the JBI Checklist for Analytical Cross-Sectional Studies (8 items) was employed.^18^ For the cohort studies, the JBI Checklist for Cohort Studies (11 items) was used.^18^, ^19^ Each item was answered as "Yes" (Y), "No" (N), "Unclear" (U), or "Not applicable" (NA). Based on previously published thresholds, cross-sectional studies scoring ≥ 6 out of 8 were classified as high quality, 5 out of 8 as moderate, and ≤ 4 out of 8 as low quality.^20^ Cohort studies scoring ≥ 8 out of 11 were classified as high quality, 6–7 out of 11 as moderate quality, and ≤ 5 out of 11 as low quality.^21^ Four independent assessors (RDT, NTG, MGF, and YMM) evaluated each study. Discrepancies in quality ratings were resolved through consensus discussion with additional reviewers (TBA, ZAY, GAB, and WNM).

### Data extraction process

A standardized, pre-piloted data extraction sheet in Microsoft Excel facilitated the data collection. Two independent reviewers (TBA, YAW) extracted key variables, including author/year, study design, geographical setting, adherence measurement tool, sample size, non-adherence prevalence, and significant factors with their effect estimates. Discrepancies were resolved by consensus, and a cross-checking procedure ensured data accuracy.

### Operational definitions

Good adherence: As defined by the original studies, typically using standardized tools like the Morisky Medication Adherence Scale (MMAS−8), where a score above a defined threshold (e.g., ≥6 for MMAS−8) indicates good adherence.

Poor adherence / Non-adherence: As defined by the original studies, typically a score below a defined threshold (e.g., <6 for MMAS−8) indicating poor adherence.^22^, ^23^

Associated factors were variables reported to have a statistically significant association (p-value < 0.05) with medication non-adherence in multivariate analysis within the included studies.

Extreme age groups were defined as studies focusing exclusively on elderly patients (≥60 years of age) or exclusively on pediatric patients (≤18 years of age).^24^, ^25^

### Statistical analysis and data synthesis

The analytical phase began by transferring the dataset, which was initially compiled and organized in Microsoft Excel, into Stata version 17 for comprehensive statistical analysis. A descriptive analysis was first performed to summarize the key characteristics of the included studies, providing an overview of the literature. The core of the meta-analysis involved generating forest plots to visually present the individual study estimates and calculate the overall pooled prevalence of medication non-adherence. Prior to this, the presence and extent of statistical heterogeneity across the studies were rigorously evaluated. This was done using the Cochrane Q statistic (a measure of whether differences in results are due to chance) and the I² statistic, which quantifies the percentage of total variation across studies that is due to heterogeneity rather than chance. A high I² value indicated significant variability, suggesting that the studies were not estimating a single common effect.

Furthermore, the potential for publication bias—the tendency for positive or significant results to be published over null findings—was assessed. This was done through both a visual examination of a funnel plot for asymmetry and a statistical evaluation using Egger's regression test. Due to the significant heterogeneity confirmed by the I² statistic, a random-effects meta-analysis model, as proposed by DerSimonian and Laird, was selected for pooling the data. This model accounts for both within-study sampling error and between-study variation, providing a more conservative and generalizable estimate than a fixed-effect model. To explore the substantial heterogeneity, subgroup analyses were performed. These analyses investigated whether the effect sizes differed systematically based on specific study characteristics, namely geographic region within Africa, the specific tool used to measure adherence, and the study's sample size. Although different adherence scales have varying sensitivity and specificity, all included tools were validated instruments designed to assess medication adherence. Prior meta-analyses^26^, ^27^ have similarly combined different adherence measures, as the binary outcome (adherent vs. non-adherent) is consistently reported regardless of the specific tool used. Finally, to assess the robustness and stability of the pooled prevalence estimate, a leave-one-out sensitivity analysis was conducted. This method involved systematically removing one study at a time and recalculating the overall estimate with the remaining studies. This process verifies whether any single study exerts a disproportionate influence on the final result.

## Results

### Search outcomes

We identified 342 articles through a systematic search of electronic databases. After removing duplicates, 98 records were screened by title and abstract, leading to the exclusion of 63 that were irrelevant. The full text of the remaining 35 articles was assessed for eligibility. Of these, 16 were excluded for the following reasons: ineligible study design (n = 4),^28^, ^29^, ^30^, ^31^ incorrect patient population (n = 5),^32^, ^33^, ^34^, ^35^, ^36^ insufficient data on outcome variable (n = 2),^11^, ^37^ and because they did not report a multivariate analysis for associated factors (n = 5).^38^, ^39^, ^40^, ^41^ A total of 19 studies qualified for the systematic review. For the final meta-analysis, which required specific quantitative data, 17 studies were included,^24^, ^25^, ^42^, ^43^, ^44^, ^45^, ^46^, ^47^, ^48^, ^49^, ^50^, ^51^, ^52^, ^53^, ^54^, ^55^, ^56^ as two studies were excluded due to data that could not be statistically pooled.^57^, ^58^ The complete selection process is illustrated in Fig. 1.

**Fig. 1:**
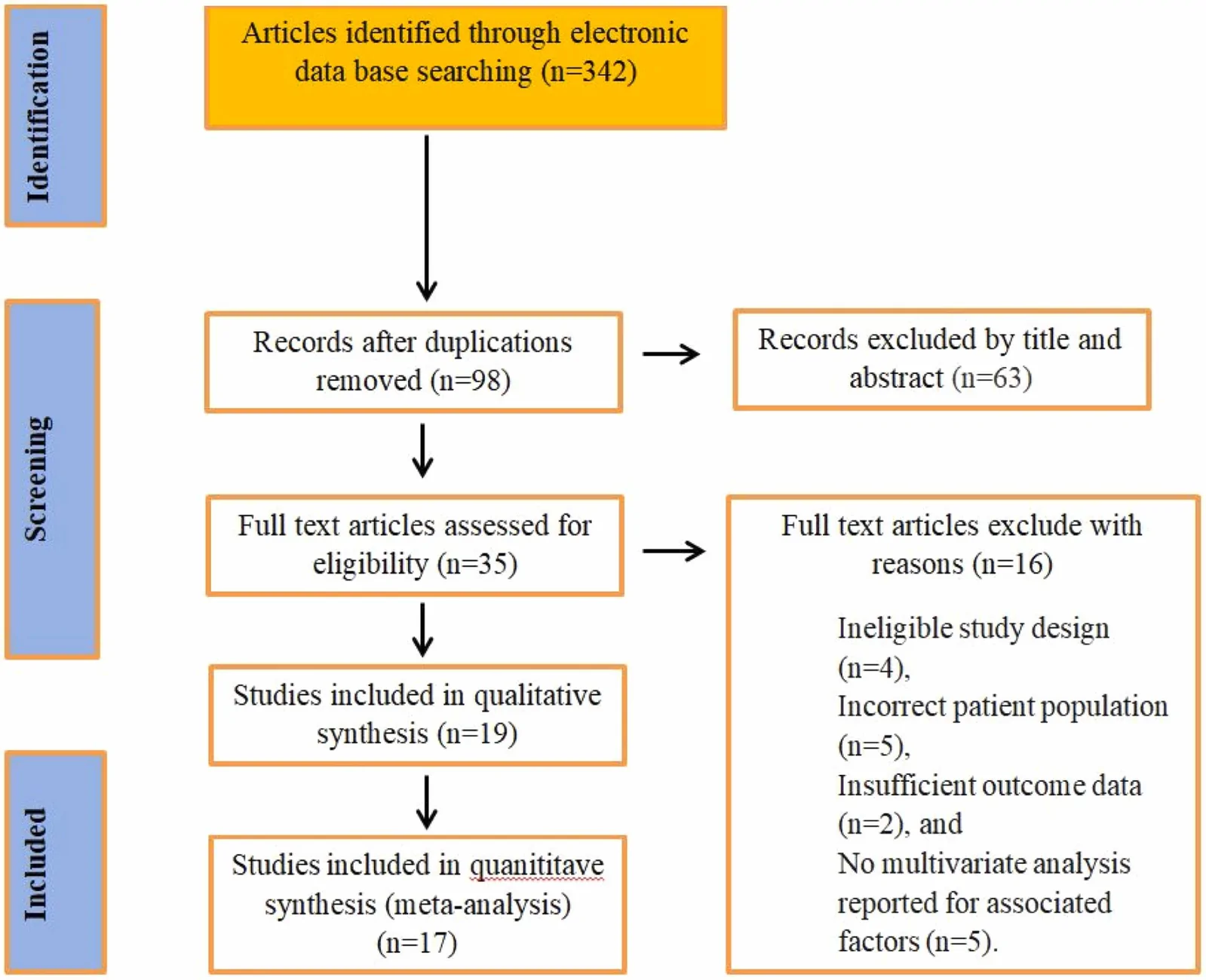
PRISMA flow diagram.

### General characteristics of included studies

This systematic review and meta-analysis included seventeen studies published between 2010 and 2025, with a total sample size of 5228 heart failure patients. The studies were conducted across various African regions, with a predominance from Eastern Africa (n = 10), followed by Western Africa (n = 3), Northern Africa (n = 3), and Southern Africa (n = 1).The sample sizes of the individual studies ranged from 103 to 603 participants. The highest prevalence of medication non-adherence was 88.4%, reported in a prospective study from Côte d′Ivoire (Western Africa), while the lowest was 1.1%, found in a cross-sectional study from Egypt (Northern Africa). Most studies utilized a cross-sectional design (n = 14), while three employed a prospective cohort design. Medication non-adherence was assessed using a variety of tools, with the Morisky Medication Adherence Scale (MMAS−8) and the Morisky Green Levin Scale (MGLS) being the most common. Characteristics of the included studies are summarized in Table 1.

**Table 1:** Characteristics of the studies included in this systematic review and meta-analysis on medication non-adherence and its associated factors among heart failure patients in Africa.

Authors	Year of Publication	Study design	Assessment Tool	Region	Study population	Sample size	Prevalence	Joanna Briggs Institute (JBI) critical appraisal tools score
Tarekegn et al.	2025	Cross-sectional	Medication Adherence Rating Scale (MARS−5)	Ethiopia	Adult	360	17.7	7
Azizew et al.	2025	Cross-sectional	Pill count	Ethiopia	Children	203	23.15	7
Dagnew et al.	2024	Cross-sectional	Adherence in Chronic Diseases Scale (ACDS)	Ethiopia	Adult	336	29.17	7
Tsige et al.	2024	Cross-sectional	Morisky Green Levin Medication Adherence Scale (MGLS)	Ethiopia	Adult	344	39.1	7
Hussein et al.	2024	Cross-sectional	Morisky Medication Adherence Scale (MMAS−8)	Ethiopia	Adult	603	44	7
Seid et al.	2023	Cross-sectional	Medication Adherence Rating Scale (MARS−5)	Ethiopia	Adult	245	23.7	7
Damte et al.	2022	Cross-sectional	Morisky Medication Adherence Scale (MMAS−8)	Ethiopia	Adult	385	51.4	8
Seid et al.	2020	Cross-sectional	Morisky Green Levin Medication Adherence Scale (MGLS)	Ethiopia	Adult	423	26.2	8
Fetensa et al.	2019	Cross-sectional	Morisky Green Levin Medication Adherence Scale (MGLS)	Ethiopia	Adult	424	35.14	7
Ruf et al.	2010	Cross-sectional	Pill count	South Africa	Adult	200	29	6
Awad et al.	2017	Cross-sectional	Morisky Medication Adherence Scale (MMAS−8)	Sudan	Adult	433	51	5
Pallangyo et al.	2018	prospective cohort study	Morisky Medication Adherence Scale (MMAS−8)	Tanzania	Adult	419	74.7	8
Ale et al.	2021	Cross-sectional	Medication Adherence Rating Scale (MARS−5)	Nigeria	Adult	202	69.3	6
Yayehd et al.	2012	Prospective Cohort Study	Disease-Specific Questionnaire (Medication, Diet, Knowledge)	Togo	Adult	103	74.7	7
N'Cho-Mottoh et al.	2025	Prospective Observational Study	Revised Heart Failure Compliance Questionnaire	Côte d′Ivoire	Adult	121	88.4	8
Shoulkamy et al.	2018	Cross-sectional	Revised Heart Failure Compliance Questionnaire	Egypt	Adult	280	1.1	6
Ragbaoui et al., 2017	2017	Cross-sectional	CARDIA-Questionary	Morocco	Adult	147	16.7	5

### Heterogeneity and publication bias

To determine the pooled prevalence of medication non adherence among heart failure patients, a random-effects meta-analysis was employed, specifically utilizing the Der Simonian and Laird method for combining study effects. The I² inconsistency statistic was rigorously applied to assess the heterogeneity among the included studies. The heterogeneity of the twenty articles included in the current systematic review and meta-analysis was high, as shown by the test statistics (I² = 97.7%, *p* value < 0.001). A visual assessment of the funnel plot was conducted to explore publication bias, and the symmetrical distribution observed suggests the absence of such bias. Furthermore, Egger’s test was performed to provide an objective evaluation (*p* = 0.246) (Table 2), confirming no evidence of publication bias. The funnel plot is shown in Fig. 2.

**Table 2:** Egger’s test of the medication non-adherence and its associated factors among heart failure patients in Africa.

Std_Eff	Coef.	Std. Err.	t	*P* > |t|	[95% Conf. Interval]
slope	−10.1552	7.018663	−1.45	0.169	−25.11512, 4.804731
bias	20.12842	3.824043	5.26	0.246	11.97766, 28.27917

**Fig. 2:**
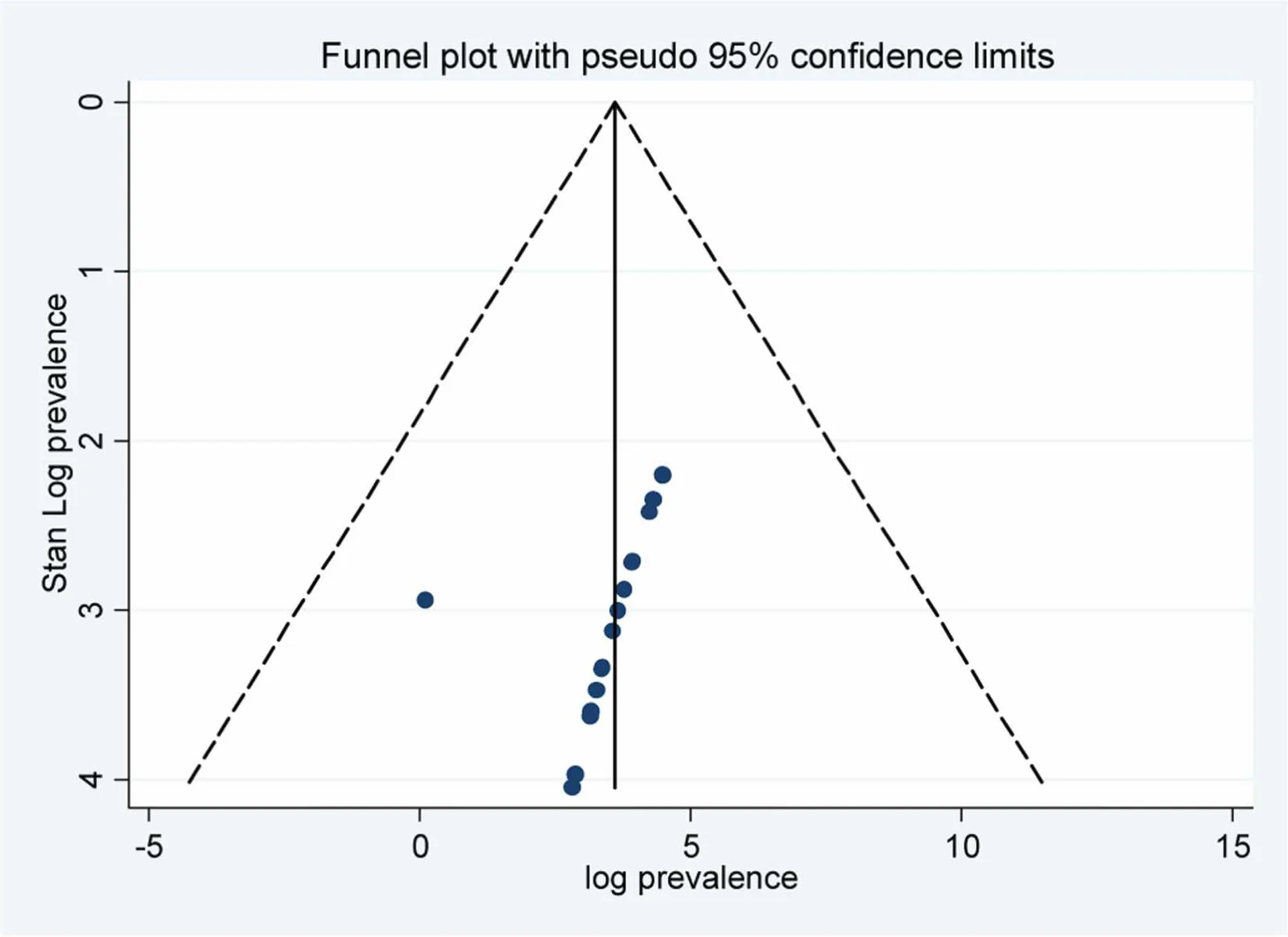
A funnel plot test meta-analysis shows an absence of publication bias.

### Subgroup analysis

Subgroup analysis was conducted based on region, assessment tool, study design, and study population to explore the sources of the significant heterogeneity (I² = 99.7%, *p* < 0.001) identified in the primary analysis.

By Region: The analysis revealed profound geographical disparities. The prevalence was highest in Western Africa at 77.59% (95% CI: 65.20–89.98), followed by Eastern Africa at 37.77% (95% CI: 27.34–48.20). The prevalence was substantially lower in Southern Africa (29.00%; 95% CI: 22.71–35.29) and Northern Africa (8.61%; 95% CI: −6.67–23.89).

By Assessment Tool: The instrument used to measure adherence was a major source of variation. Studies using the Morisky Medication Adherence Scale (MMAS−8) reported the highest prevalence at 55.29% (95% CI: 41.28–69.29). In contrast, those using the Morisky Green Levin Scale (MGLS) reported 33.37% (95% CI: 25.77–40.97), and studies categorized as "Others" (pill count and Adherence in Chronic Disease Scale (ACDS)) reported the most consistent and lowest prevalence at 27.22% (95% CI: 23.38–31.06), with low heterogeneity (I² = 28.9%). Furthermore, subgroup analysis by assessment tool revealed no statistically significant difference in pooled prevalence estimates, supporting the validity of combining these measures.

By Study Design: A marked difference was observed based on methodology. Prospective cohort studies yielded a significantly higher pooled prevalence of 79.37% (95% CI: 69.93–88.81) compared to cross-sectional studies at 32.58% (95% CI: 20.51–44.66).

By Study Population: The target population also influenced the results. Studies focusing on general adult populations reported a prevalence of 44.62% (95% CI: 33.69–55.55), while those restricted to extreme age groups (children and the elderly) reported a much lower prevalence of 11.94% (95% CI: −9.67–33.54). The overall results of the subgroup analysis are presented in Table 3.

**Table 3:** Subgroup analysis of medication non-adherence and its associated factors among heart failure patients in Africa.

Variables	Subgroup	Number of studies	Prevalence (95% CI)	I2%	*p-*value
Region	Eastern Africa	11	37.77 (27.34, 48.20)	98.2	0.000
	Southern Africa	1	29.00 (22.71, 35.29)	-	-
	Western Africa	3	77.59 (65.20, 89.98)	90.2	0.000
	Northern Africa	2	8.61 (−6.67, 23.89)	96.0	0.000
Assessment Tool	Morisky Green Levin Medication Adherence Scale (MGLS)	3	33.37 (25.77, 40.97)	87.7	0.000
	Morisky Medication Adherence Scale (MMAS−8)	4	55.29 (41.28, 69.29)	97.5	0.000
	Medication Adherence Rating Scale (MARS−5)	3	36.82 (8.01, 65.62)	98.9	0.000
	Others*	3	27.22 (23.38, 31.06)	28.9	0.245
	Not Available**	4	45.15 (−0.61, 90.92)	99.7	0.000
Study design	Cross-sectional	14	32.58 (20.51, 44.66)	99.2	0.000
	Prospective Cohort	3	79.37 (69.93, 88.81)	87.1	0.000
Study population	Adults	15	44.62 (33.69, 55.55)	98.6	0.000
	Extreme age groups***	2	11.94 (−9.67, 33.54)	98.1	0.000

*Adherence in Chronic Disease Scale (ACDS) and Pill count

**Not available indicates that the primary study did not report the specific assessment tool used.

***Extreme age groups were defined as studies focusing exclusively on elderly patients (≥60 years) or pediatric patients (≤18 years), consistent with the populations examined in the primary studies.

### Sensitivity analysis

Results of the leave-one-out sensitivity analysis of the pooled prevalence for each excluded study remained within the CI (ranging between 37.83% and 43.28%) of the analysis that combined all the studies, as presented in Table 4a. This suggests that the overall estimate is robust and not significantly affected by any single study. To assess the influence of potential outliers, a leave-one-out meta-analysis was performed after excluding the studies reporting the highest (88.4%) and lowest (1.1%) prevalence values. As shown in Table 4b, the pooled prevalence estimates after sequential omission of each study ranged from 37.74% to 41.94%, all within the 95% confidence interval of the overall pooled estimate (40.27%; 30.66–49.88). Heterogeneity remained high (I² = 97.3–98.6%). These findings confirm that no single study disproportionately influenced the pooled estimate, supporting the robustness of the meta-analysis results.

**Table 4a:** Sensitivity analysis of medication non-adherence and its associated factors among heart failure patients in Africa included studies in this meta-analysis and systematic review.

Studies omitted	Year of publication	Estimate prevalence (95% CI)	Heterogeneity	
			I^2^	*p*-value
Tarekegn et al.	2025	42.26 (27.28, 57.23)	99.8	< 0.001
Azizew et al.	2025	41.91 (27.28, 56.54)	99.8	< 0.001
Dagnew et al.	2024	41.53 (26.80, 56.26)	99.8	< 0.001
Tsige et al.	2024	40.91 (26.32, 55.50)	99.8	< 0.001
Hussein et al.,	2024	40.61 (25.96, 55.25)	99.8	< 0.001
Seid et al.	2023	41.87 (27.19, 56.56)	99.8	< 0.001
Damte et al.	2022	40.14 (25.76, 54.52)	99.8	< 0.001
Seid et al.	2020	41.72 (26.83, 56.61)	99.8	< 0.001
Fetensa et al.	2019	41.16 (26.43, 55.89)	99.8	< 0.001
Ruf et al.	2010	41.54 (26.97, 56.11)	99.8	< 0.001
Awad et al.	2017	40.17 (25.77, 54.56)	99.8	< 0.001
Pallangyo et al.	2018	38.67 (25.45, 51.88)	99.7	< 0.001
Ale et al.	2021	39.03 (24.99, 53.07)	99.8	< 0.001
Yayehd et al.	2012	38.71 (24.61, 52.81)	99.8	< 0.001
N'Cho-Mottoh et al.	2025	37.83 (24.54, 51.11)	99.8	< 0.001
Shoulkamy et al.	2018	43.28 (32.77, 53.79)	99.8	< 0.001
Ragbaoui et al.	2017	42.31 (27.70, 56.91)	99.8	< 0.001

**Table 4b:** Leave-one-out sensitivity analysis of medication non-adherence prevalence among heart failure patients in Africa after exclusion of extreme outlier studies (1.1% and 88.4% prevalence).

Studies omitted	Year of Publication	Estimate prevalence (95% CI)	Heterogeneity	
			I^2^	*p*-value
Tarekegn et al.	2025	41.89 (32.24, 51.54)	98.0	< 0.001
Azizew et al.	2025	41.48 (31.46, 51.51)	98.2	< 0.001
Dagnew et al.	2024	41.07 (30.84, 51.29)	98.3	< 0.001
Tsige et al.	2024	40.35 (30.05, 50.65)	98.1	< 0.001
Hussein et al.	2024	40.00 (29.51, 50.50)	98.3	< 0.001
Seid et al.	2023	41.45 (31.40, 51.50)	98.6	< 0.001
Damte et al.	2022	39.47 (29.31, 49.63)	98.3	< 0.001
Seid et al.	2020	41.28 (31.08, 51.48)	98.2	< 0.001
Fetensa et al.	2019	40.639 (30.26, 51.02)	98.3	< 0.001
Ruf et al.	2010	41.07 (30.95, 51.18)	98.3	< 0.001
Awad et al.	2017	39.50 (29.30, 49.69)	98.4	< 0.001
Pallangyo et al.	2018	37.74 (29.58, 45.90)	97.3	< 0.001
Ale et al.	2021	38.21 (28.63, 47.79)	98.1	< 0.001
Yayehd et al.	2012	37.88 (28.27, 47.50)	98.2	< 0.001
Ragbaoui et al.	2017	41.94 (32.10, 51.77)	98.2	< 0.001

### Pooled prevalence of medication non-adherence

The combined prevalence of medication non-adherence across the seventeen studies was 40.8%, with a 95% CI ranging from 26.89% to 54.72% using the random effect model. The forest plot in Fig. 3 below depicts the pooled prevalence.

**Fig. 3:**
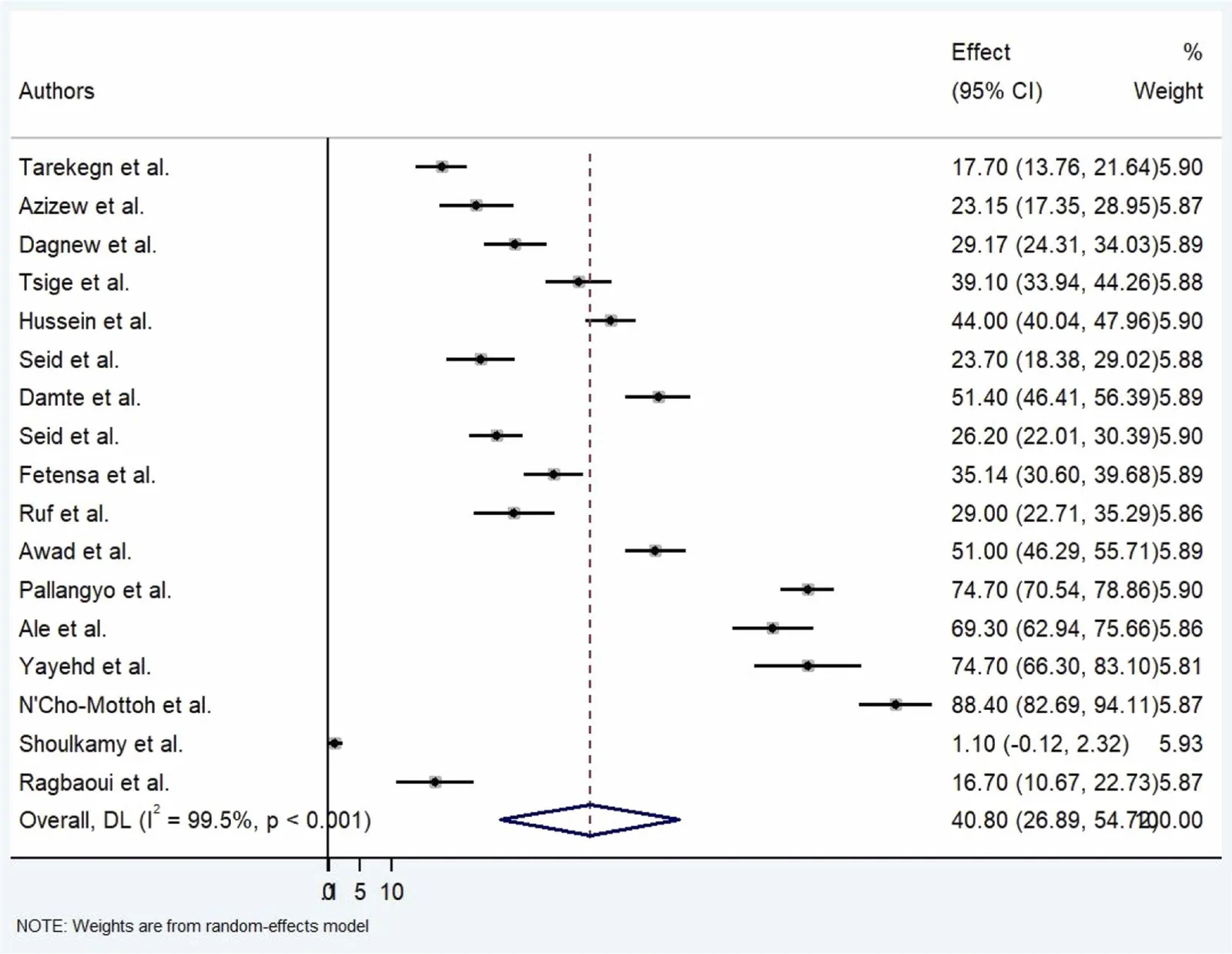
Forest plot showing the pooled prevalence of medication non-adherence among heart failure patients in Africa.

### Factors associated with medication non-adherence

Lack of health insurance, polypharmacy, rural residence, and the presence of comorbidities were identified as factors significantly associated with medication non-adherence among heart failure patients in Africa. The meta-analysis revealed that patients without health insurance had approximately five times higher odds of non-adherence than those with insurance (OR: 5.13, 95% CI: 2.03–12.92). Similarly, those with polypharmacy had a 2.58 times higher likelihood of non-adherence (OR: 2.58, 95% CI: 2.00–3.33) than those without. Patients residing in rural areas faced more than double the odds (OR: 2.19, 95% CI: 1.33–3.60) compared to their urban counterparts. Finally, the presence of comorbidities was associated with twice the odds of non-adherence (OR: 2.01, 95% CI: 1.42–2.83). Fig. 4 summarises this.

**Fig. 4:**
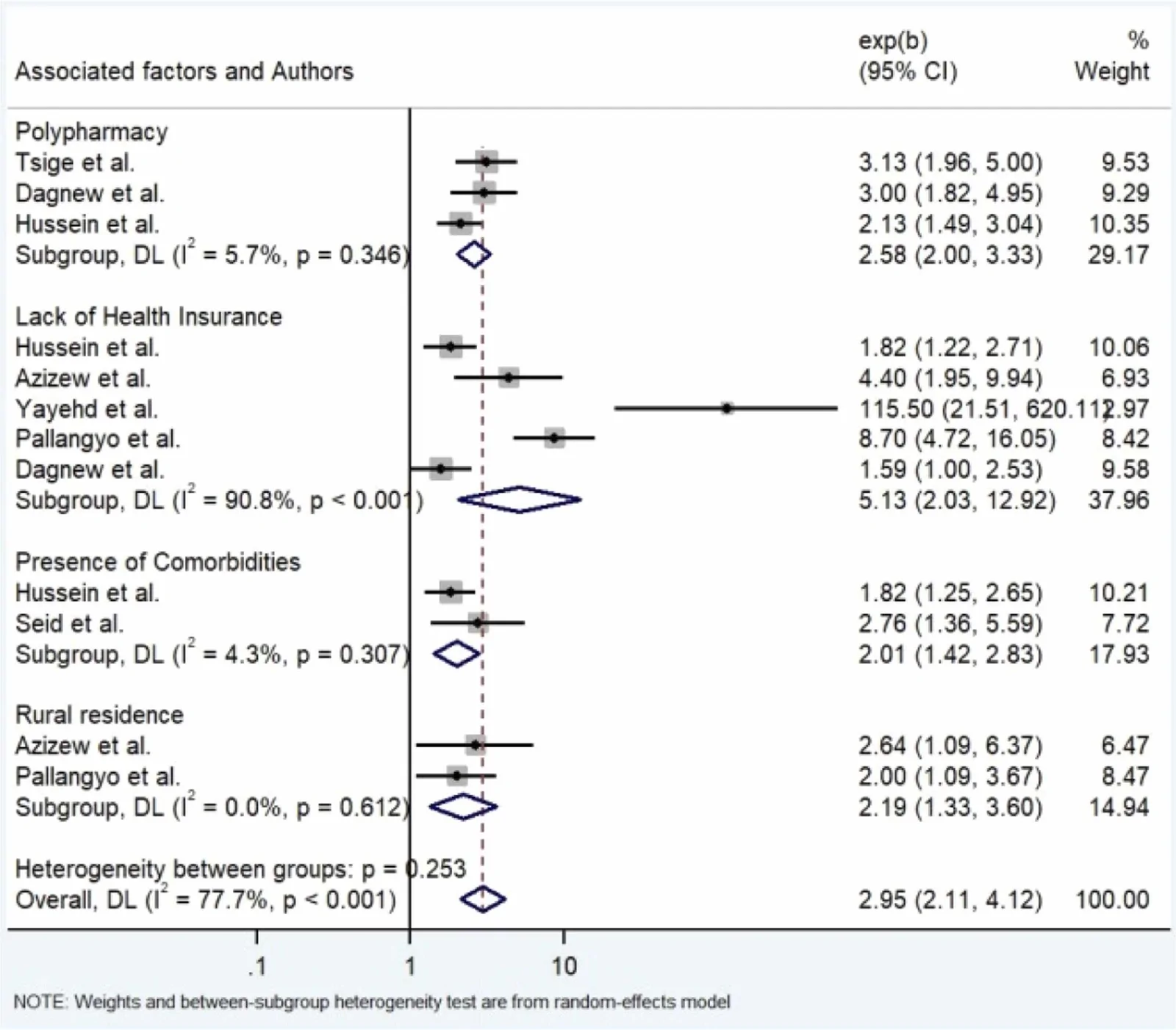
The forest plot shows associated factors of medication non-adherence among heart failure patients in Africa.

### Quality of the included studies

Among the fourteen cross-sectional studies, ten scored 7 out of 8 (high quality), two scored 8 out of 8 (high quality), two scored 6 out of 8 (high quality), and two scored 5 out of 8 (moderate quality). All fourteen cross-sectional studies were therefore of moderate to high quality. Among the three cohort studies, two scored 8 out of 11 (high quality) and one scored 7 out of 11 (moderate quality). Full quality assessment details are provided in Supplementary Material 2 (Tables S2a and S2b).

## Discussion

This systematic review and meta-analysis synthesizes evidence from seventeen primary studies across Africa, revealing a pooled medication non-adherence prevalence of 40.8% (95% CI: 26.89–54.72) among heart failure patients. Obtaining a comprehensive pooled prevalence estimate is challenging due to limited studies in many African regions; however, the rate identified in this analysis falls within—though at the lower end of—global estimates, which range from 40 to 60% and reach 72.9% in the United States^59^, ^60^, ^61^, ^62^, ^63^ and 60% in the Middle East.^27^ This apparent discrepancy should not be interpreted as superior adherence in African contexts but may reflect critical methodological and contextual differences. In resource-constrained settings, the clinical presentation of heart failure is often more severe and occurs at a younger age.^64^ The palpable severity of symptoms in advanced cases may motivate adherence, as patients directly associate medication intake with symptomatic relief and immediate survival.^65^ Unlike healthcare systems in the US, where medication costs are primarily covered by insurance, African healthcare systems rely heavily on out-of-pocket payments, which directly impacts affordability and adherence.^66^ Additionally, differences in healthcare infrastructure, medication supply chain reliability, and availability of pharmacy services distinguish African settings from Middle Eastern or Western contexts.^67^ Notably, adherence rates varied widely across studies (1.1–88.4%), highlighting the complex interplay of contributing factors and the need for deeper investigation—a pattern also observed in Middle Eastern studies.^27^ Interpreting these results within the WHO’s five dimensions of medication adherence provides a comprehensive framework for understanding the multifactorial influences on treatment behaviors. The considerable heterogeneity (I² = 99.7%) reflects both methodological diversity and genuine contextual differences across African health systems. Pronounced regional disparities were observed, with the highest non-adherence in Western Africa (77.59%), followed by Eastern (42.03%), Southern (28.79%), and Northern Africa (21.30%). These differences likely arise from variations in healthcare infrastructure, economic conditions, medication availability, and health insurance coverage. Furthermore, rural-urban disparities and patient demographic characteristics may have contributed to the observed heterogeneity, but these factors were not consistently reported in the included primary studies, limiting formal investigation. Several African-specific factors may potentially influence medication adherence, though evidence remains limited. Traditional medicine use may reduce reliance on conventional medications, while strong family support structures may either facilitate or hinder adherence depending on individual family dynamics.^68^ Medication sharing practices, reported in some African communities, could potentially contribute to treatment interruption.^68^ However, these factors are primarily based on observational evidence and require further investigation. Therefore, these potential influences should be interpreted with caution. The extreme heterogeneity observed may also be attributed to differences in healthcare system structures, economic conditions, rural-urban disparities, and patient demographic characteristics across the included studies, which were not consistently reported in primary studies.^32^, ^69^ A strong association was identified between lack of health insurance and non-adherence (OR = 5.13), emphasizing the vital role of financial protection in medication access—a finding especially relevant in settings reliant on out-of-pocket payments.

The therapy-related dimension of the WHO framework is reinforced by the significant association between polypharmacy and non-adherence (OR = 2.58), a critical concern in resource-limited settings where medication reconciliation and pharmacist-led management are often unavailable. Similarly, the condition-related dimension is substantiated by the effect of comorbidities (OR = 2.01), which amplify both clinical and economic burdens for patients managing multiple treatments.

In comparison with global literature, African findings share commonalities with Middle East study,^27^ particularly regarding the influence of financial barriers and polypharmacy. However, the effect of financial constraints appears more pronounced in African settings. Additionally, while studies in the Middle East report variable impacts of education and socioeconomic status,^27^ this review found financial barriers to be more consistently significant than educational factors in the African context. Methodological factors significantly influence the interpretation of these findings. The substantial discrepancy in adherence rates between prospective cohort studies (79.37%) and cross-sectional designs (36.27%) indicates that study design substantially affects adherence measurement. This pattern suggests that single-point assessments may systematically underestimate true non-adherence rates, highlighting the importance of longitudinal approaches in adherence research.^70^ Furthermore, notable variations emerged across assessment methodologies. Studies utilizing the Morisky Medication Adherence Scale−8 (MMAS−8) reported the highest non-adherence prevalence (48.49%), followed by those employing the Morisky Green Levine Scale (MGLS) (34.80%), while pill count methods yielded the lowest estimates (24.59%). This hierarchical pattern demonstrates how measurement tools substantially influence adherence classification. The MMAS−8's comprehensive 8-item structure appears particularly sensitive in detecting both intentional and unintentional non-adherence behaviors common in resource-constrained settings.^22^ Its multidimensional assessment likely captures medication-taking nuances that other methods may miss. The MGLS, while also a self-report instrument, demonstrated intermediate detection rates, potentially reflecting its distinct question structure and scoring algorithm.^71^ Pill count methods, providing objective behavioral data rather than self-reported information, yielded the lowest estimates.^72^ However, they may underestimate true non-adherence in African contexts due to several factors: the "white coat effect" (improved adherence preceding clinic visits), limited healthcare access preventing regular assessments, and medication sharing within communities—a common practice in resource-limited settings that pill counts cannot capture.^73^, ^74^ These methodological variations underscore the urgent need for standardized, validated measurement approaches specifically adapted for African healthcare contexts to ensure more accurate and comparable adherence assessment.

Based directly on the specific findings of this meta-analysis, we recommend targeted region-specific and factor-driven interventions. Given that Western Africa had the highest pooled non-adherence prevalence (77.59%)—more than double that of Eastern Africa (37.77%) and nine times that of Northern Africa (8.61%)—intervention efforts should be urgently prioritized in Western African countries. Regarding financial protection, lack of health insurance was the strongest predictor of non-adherence (OR = 5.13), therefore African governments should prioritize expanding health insurance coverage specifically for heart failure patients. For therapy-related barriers, polypharmacy was significantly associated with non-adherence (OR = 2.58), supporting the implementation of regular medication review services and fixed-dose combination therapies to reduce pill burden. Given that rural residence was associated with higher non-adherence odds (OR = 2.19), digital health solutions such as mobile health platforms for medication reminders and telemedicine consultations should be directly targeted to rural patients who face geographic barriers to healthcare access. Additionally, the presence of comorbidities was associated with non-adherence (OR = 2.01), necessitating integrated heart failure education programs that simultaneously manage multiple chronic conditions. Finally, given that assessment tool choice substantially influenced reported prevalence (55.29% for MMAS−8 vs. 27.22% for pill count), future research should adopt validated, standardized adherence measurement tools appropriate for African settings to reduce measurement heterogeneity.

This review offers several strengths, including comprehensive coverage of multiple African regions, rigorous adherence to PRISMA guidelines, and use of the WHO adherence framework for contextual analysis. The inclusion of both English and French literature reduces language bias, while quality assessment using the Joanna Briggs Institute (JBI) critical appraisal tools ensure methodological rigor. Sensitivity analysis confirmed that no single study disproportionately influenced the pooled estimate, despite the wide prevalence range (1.1–88.4%) observed across African regions. This indicates that the substantial variation reflects true regional differences in medication non-adherence across African healthcare settings rather than the influence of isolated outlier studies.

However, several limitations merit consideration. The reliance on observational studies limits causal inferences, while variability in adherence measurement tools across studies may affect comparability. Publication bias remains possible despite statistical tests showing non-significance. Additionally, the exclusion of non-published studies and those in other languages might overlook relevant data. The meta-analysis of associated factors was limited by few studies per factor (2–6 studies), resulting in wide confidence intervals (e.g., lack of health insurance) and imprecision. These findings should therefore be interpreted with caution, and future primary studies—including larger multi-center research—are needed to confirm these associations once such data become available. Given the predominance of studies from Eastern Africa (particularly Ethiopia) with underrepresentation of Western, Northern, and Southern Africa, we recommend that future primary research be prioritized in these underrepresented regions to enable more comprehensive continent-wide estimates.

## Conclusions

This comprehensive review demonstrates that medication non-adherence among heart failure patients in Africa is a complex, multidimensional problem with a pooled prevalence of 41.88%. The extreme regional variations and strong associations with financial barriers, polypharmacy, and comorbidities underscore the need for context-specific interventions addressing all five WHO dimensions of medication adherence. The findings highlight both the urgency of this public health challenge and the opportunity for significant improvement through coordinated, evidence-based interventions. Future efforts must focus on implementing comprehensive strategies that address financial protection, therapy simplification, comorbidity management, and healthcare system strengthening while developing standardized approaches to adherence measurement. Through such multifaceted, context-sensitive approaches, African healthcare systems can significantly improve medication adherence and clinical outcomes for heart failure patients across the continent.

## CRediT authorship contribution statement

**Temesgen Getachew Shiferaw:** Writing – review & editing, Visualization, Validation, Investigation. **Hana Girma Abera:** Writing – review & editing, Validation, Investigation, Formal analysis. **Hailemaryam Yohannes Asefa:** Writing – review & editing, Validation, Methodology, Investigation. **Diro Ajema Cheka:** Writing – review & editing, Validation, Investigation, Formal analysis. **Zena Admasu Yferu:** Writing – review & editing, Validation, Methodology, Investigation. **Tadele Mesfin Demelash:** Writing – review & editing, Validation, Investigation, Formal analysis. **Bezawit Dereje Tilahun:** Writing – review & editing, Visualization, Supervision, Data curation. **Leul Wondemu Sebsebe:** Writing – review & editing, Validation, Investigation. **Yilkal Abebaw Wassie:** Writing – review & editing, Visualization, Supervision, Methodology, Investigation, Data curation. **Redeat Dereje Tilahun:** Writing – review & editing, Validation, Methodology, Investigation. **Wasihun Nigdu Mengestu:** Writing – review & editing, Validation, Methodology, Investigation. **Melake Getahun Fikru:** Writing – review & editing, Validation, Methodology, Investigation. **Nardos Teshome Gurmu:** Writing – review & editing, Validation, Investigation, Formal analysis. **Abera Girma Beyena:** Writing – review & editing, Validation, Investigation, Formal analysis. **Gebremariam Wulie Geremew:** Writing – review & editing, Validation, Investigation. **Tesfaye Birhanu Abebe:** Writing – review & editing, Writing – original draft, Methodology, Formal analysis, Data curation, Conceptualization. **Yohana Moges Mehari:** Writing – review & editing, Validation, Investigation, Formal analysis. **Teklestadik Teklesillasie Alemayehu:** Writing – review & editing, Supervision, Methodology, Formal analysis, Conceptualization.

## Consent for publication

Not applicable.

## Ethical approval

Not applicable.

## Declaration of Generative AI and AI-assisted technologies in the writing process

No AI tools were used in manuscript writing.

## Funding

This research did not receive any specific grant from funding agencies in the public, commercial, or not-for-profit sectors.

## Declaration of Competing Interest

The authors have declared that they have no competing interests.

## References

[1] Minja N.W., Nakagaayi D., Aliku T. (2022). Cardiovascular diseases in Africa in the twenty-first century: gaps and priorities going forward. Front Cardiovasc Med.

[2] Savarese G., Becher P.M., Lund L.H., Seferovic P., Rosano G.M., Coats A.J. (2022). Global burden of heart failure: a comprehensive and updated review of epidemiology. Cardiovasc Res.

[3] Chauke G.D., Nakwafila O., Chibi B., Sartorius B., Mashamba-Thompson T. (2022). Factors influencing poor medication adherence amongst patients with chronic disease in low-and-middle-income countries: A systematic scoping review. Heliyon.

[4] Vrijens B., De Geest S., Hughes D.A. (2012). A new taxonomy for describing and defining adherence to medications. Br J Clin Pharmacol.

[5] Fernandez-Lazaro C.I., Adams D.P., Fernandez-Lazaro D., Garcia-González J.M., Caballero-Garcia A., Miron-Canelo J.A. (2019). Medication adherence and barriers among low-income, uninsured patients with multiple chronic conditions. Res Social Adm Pharm.

[6] Luce B.R., Elixhauser A. (2012).

[7] Bosworth H.B., Granger B.B., Mendys P. (2011). Medication adherence: a call for action. Am Heart J.

[8] Van Hecke A., Heinen M., Fernández-Ortega P. (2017). Systematic literature review on effectiveness of self-management support interventions in patients with chronic conditions and low socio-economic status. J Adv Nurs.

[9] Jin J., Sklar G.E., Min Sen Oh V., Chuen Li S. (2008). Factors affecting therapeutic compliance: a review from the patient’s perspective. Ther Clin risk Manag.

[10] Alemayehu T.T., Geremew G.W., Tegegne A.A. (2025). Drug-drug interaction among elderly patients in Africa: a systematic review and meta-analysis. BMC Pharmacol Toxicol.

[11] Kasahun A.E., Sendekie A.K., Mekonnen G.A., Sema F.D., Kemal L.K., Abebe R.B. (2022). Impact of personal, cultural and religious beliefs on medication adherence among patients with chronic diseases at University Hospital in Northwest Ethiopia. Patient Prefer adherence.

[12] Banerjee A., Khandelwal S., Nambiar L. (2016). Health system barriers and facilitators to medication adherence for the secondary prevention of cardiovascular disease: a systematic review. Open Heart.

[13] Abebe T.B., Bedada G.J., Fantu Y.T. (2025). Unilateral global renal infarction in a patient with ischemic stroke and hypertensive heart disease in a resource limited setting: a case report. BMC Nephrol.

[14] Bansilal S., Castellano J.M., Garrido E. (2016). Assessing the impact of medication adherence on long-term cardiovascular outcomes. J Am Coll Cardiol.

[15] Religioni U., Barrios-Rodríguez R., Requena P., Borowska M., Ostrowski J. (2025). Enhancing therapy adherence: impact on clinical outcomes, healthcare costs, and patient quality of life. Medicina.

[16] Shanbhag D., Graham I.D., Harlos K. (2018). Effectiveness of implementation interventions in improving physician adherence to guideline recommendations in heart failure: a systematic review. BMJ Open.

[17] Page M.J., McKenzie J.E., Bossuyt P.M. (2021). The PRISMA 2020 statement: an updated guideline for reporting systematic reviews. bmj.

[18] Barker T.H., Stone J.C., Sears K. (2023). Revising the JBI quantitative critical appraisal tools to improve their applicability: an overview of methods and the development process. JBI Evid Synth.

[19] Munn Z., Barker T.H., Moola S. (2020). Methodological quality of case series studies: an introduction to the JBI critical appraisal tool. JBI Evid Synth.

[20] Shi J., Gao Y., Zhao L. (2020). Prevalence of delirium, depression, anxiety, and post-traumatic stress disorder among COVID-19 patients: protocol for a living systematic review. Syst Rev.

[21] Borges Migliavaca C., Stein C., Colpani V. (2020). How are systematic reviews of prevalence conducted? A methodological study. BMC Med Res Methodol.

[22] Tan X., Patel I., Chang J. (2014). Review of the four item Morisky medication adherence scale (MMAS-4) and eight item Morisky medication adherence scale (MMAS-8). Innovations Pharm.

[23] Moon S.J., Lee W.-Y., Hwang J.S., Hong Y.P., Morisky D.E. (2017). Accuracy of a screening tool for medication adherence: A systematic review and meta-analysis of the Morisky Medication Adherence Scale-8. PloS One.

[24] Shoulkamy T.A., Hassanen R.H., Abozeid H.A. (2018). Compliance to Therapeutic regimen Among geriatric Patients with Heart Failure at Assiut City Hospitals. Assiut Sci Nurs J.

[25] Azizew A., Dubie A.G., Zamanuel T.G., Genet G.B. (2025). Medication adherence among children with heart failure at the University of Gondar Comprehensive Specialized Hospital Gondar Northwest Ethiopia. Sci Rep.

[26] Oosterom-Calo R., Van Ballegooijen A., Terwee C. (2013). Determinants of adherence to heart failure medication: a systematic literature review. Heart Fail Rev.

[27] Alfaqeeh M., Abdullah R., Zakiyah N. (2025). Medication adherence and contributing factors in patients with heart failure within the middle east: a systematic review. Glob Heart.

[28] Wondesen A., Berha A.B., Woldu M., Mekonnen D., Engidawork E. (2022). Impact of medication therapy management interventions on drug therapy problems, medication adherence and treatment satisfaction among ambulatory heart failure patients at Tikur Anbessa Specialised Hospital, Addis Ababa, Ethiopia: a one-group pre–post quasi-experimental study. BMJ Open.

[29] Hassen M., Mekonnen D., Muhammed O.S. (2022). Treatment burden among patients with heart failure attending cardiac clinic of Tikur Anbessa Specialized Hospital: an explanatory sequential mixed methods study. Sci Rep.

[30] Tsige A.W., Kotiso T.A., Ayenew K.D., Ayele S.G. (2024). Pharmacist intervention to improve adherence to medication among heart failure patients at North East Ethiopia hospital. Pharmacol Res Perspect.

[31] Ahmed K.O., Eldin I.T., Yousif M. (2022). Clinical pharmacist led-educational intervention to promote medications’ adherence for Sudanese patients with heart failure: a prospective study. Adv Pharmacol Pharm.

[32] Fenta E.T., Ayal B.G., Kidie A.A. (2024). Barriers to medication adherence among patients with non-communicable disease in North Wollo Zone Public Hospitals: socio-ecologic perspective, 2023. Patient Prefer adherence.

[33] Kassaw A.T., Sendekie A.K., Minyihun A., Gebresillassie B.M. (2024). Medication regimen complexity and its impact on medication adherence in patients with multimorbidity at a comprehensive specialized hospital in Ethiopia. Front Med.

[34] Moges T.A., Dagnew S.B., Wondm S.A. (2024). Determinants of medication non-adherence among patients with chronic diseases at community pharmacy settings in South Gondar Zone, Northwest Ethiopia: a multicenter cross-sectional study. Front Public Health.

[35] Hoevelmann J., Sliwa K., Schaar J.M. (2024). Adherence to heart failure treatment in patients with peripartum cardiomyopathy. ESC Heart Fail.

[36] Nouamou I., El Mourid M., Ragbaoui Y., Habbal R. (2019). Medication adherence among elderly patients with coronary artery disease: our experience in Morocco. Pan Afr Med J.

[37] Gamal Abd EL-Razik M., Abd El-Megeed A.E.-G., Said Sabry S., Atta Mohammed W. (2025). Knowledge and practices of older adults with cardiac disease regarding medication adherence. J Nurs Sci Benha Univ.

[38] Gelchu T., Abdela J. (2019). Drug therapy problems among patients with cardiovascular disease admitted to the medical ward and had a follow-up at the ambulatory clinic of Hiwot Fana Specialized University Hospital: the case of a tertiary hospital in eastern Ethiopia. SAGE Open Med.

[39] Bekele F., Sheleme T., Tsegaye T. (2024). Prevalence and risk factors of mortality among heart failure patients in low resource setting hospitals: a multicenter prospective observational study. Front Cardiovasc Med.

[40] Dagnew S.B., Moges T.A., Tarekegn G.Y. (2025). Treatment burden and medication adherence among older patients in comprehensive specialised hospitals in the Amhara Region in Ethiopia: a multicentre, cross-sectional study. BMJ Open.

[41] Tukeni K.N., Regassa R.J., Abera E.G., Geleta M.N. (2023). The prevalence and correlates of hospital admission of patients with heart failure admitted to Jimma Medical Center, Ethiopia. PAMJ Clin Med.

[42] Tarekegn G.Y., Dagnew F.N., Moges T.A. (2025). Medication non-adherence and its predictors among chronic heart failure patients in Northwest Amhara region. Sci Rep.

[43] Dagnew S.B., Wondm S.A., Dagnew F.N., Yimer Y.S., Wondmkun Y.T., Moges T.A. (2024). Level of medication adherence and its determinants of cardiovascular disease patients attending at specialized teaching hospitals of Amhara regional state, Ethiopia: a multicenter cross-sectional study. Front Pharmacol.

[44] Tsige A.W., Endalifer B.L., Habteweld H.A., Wondmkun Y.T., Ayele S.G., Kefale B. (2024). Treatment satisfaction and medication adherence and predictors among patients with heart failure in Ethiopia: a hospital-based cross-sectional study. Front Pharmacol.

[45] Hussein D., Jima A.K., Geleta L.A. (2024). Medication adherence and associated factors among chronic heart failure patients on follow-up in north Shewa public hospitals, Oromia region, Ethiopia. BMC Cardiovasc Disord.

[46] Seid M.A., Toleha H.N., Sema F.D. (2023). Medication nonadherence and associated factors among Heart Failure patients at University of gondar comprehensive specialized hospital, Northwest Ethiopia. Int J Chronic Dis.

[47] Damte A., Ayalew Y., Dugassa B., Habte T. (2022). Factors associated with medication adherence among adult heart failure patients at selected public hospitals of addis ababa, ethiopia. A cross-sectional study. EC Nurs Healthc.

[48] Seid E., Engidawork E., Alebachew M., Mekonnen D., Berha A.B. (2020). Evaluation of drug therapy problems, medication adherence and treatment satisfaction among heart failure patients on follow-up at a tertiary care hospital in Ethiopia. PloS One.

[49] Fetensa G., Yadecha B., Tolossa T., Bekuma T.T. (2019). Medication adherence and associated factors among chronic heart failure clients on follow up Oromia region, West Ethiopia. Cardiovasc Hematol Agents Med Chem (Former Curr Med Chem-Cardiovasc Hematol Agents.

[50] Ruf V., Stewart S., Pretorius S. (2010). Medication adherence, self-care behaviour and knowledge on heart failure in urban South Africa: the Heart of Soweto study: cardiovascular topics. Cardiovasc J Afr.

[51] Awad A., Osman N., Altayib S. (2017). Medication adherence among cardiac patients in Khartoum State, Sudan: a cross-sectional study. Cardiovasc J Afr.

[52] Pallangyo P., Millinga J., Bhalia S. (2020). Medication adherence and survival among hospitalized heart failure patients in a tertiary hospital in Tanzania: a prospective cohort study. BMC Res Notes.

[53] Ale O.K., Busari A.A., Irokosu E.S., Akinyede A.A., Usman S.O., Olayem S.O. (2021). Medication nonadherence in Nigerian heart failure patients: a cross sectional study. J Clin Sci.

[54] Yayehd, Damorou K., Mottoh F. (2013). Annales de cardiologie et d′angéiologie.

[55] N'Cho-Mottoh M., Boka B., Yayehd K. (2015). Assessment of treatment adherence among black Africans with heart failure. Médecine Et Sté Trop.

[56] Ragbaoui Y., Nouamou I., Hammiri A., Habbal R. (2017). Predictive factors of medication adherence in patients with chronic heart failure: Morocco's experience. Pan Afr Med J.

[57] Bhagat K., Mazayi-Mupanemunda M. (2001). Compliance with medication in patients with heart failure in Zambabwe. East Afr Med J.

[58] Kimani L.M., Karimi P.N., Opanga S.A., Bosire K.O. (2016). Treatment of chronic heart failure in adults at a referral hospital in Kenya: adverse drug reactions and determinants of adherence. Afr J Pharmacol Ther.

[59] Wu J.-R., Moser D.K., Lennie T.A., Burkhart P.V. (2008). Medication adherence in patients who have heart failure: a review of the literature. Nurs Clin North Am.

[60] Fitzgerald A.A., Powers J.D., Ho P.M. (2011). Impact of medication nonadherence on hospitalizations and mortality in heart failure. J Card Fail.

[61] Ruppar T.M., Delgado J.M., Temple J. (2015). Medication adherence interventions for heart failure patients: A meta-analysis. Eur J Cardiovasc Nurs.

[62] Aggarwal B., Pender A., Mosca L., Mochari-Greenberger H. (2015). Factors associated with medication adherence among heart failure patients and their caregivers. J Nurs Educ Pract.

[63] Mentz R.J., Greiner M.A., Muntner P. (2018). Intentional and unintentional medication non-adherence in African Americans: insights from the Jackson Heart Study. Am Heart J.

[64] Iloh G., Amadi C., Amadi A. (2018). Epidemiology of cardiovascular emergencies in the adult emergency department of a tertiary hospital in a resource-constrained setting of south-eastern Nigeria. J Adv Med Med Res.

[65] Weingart S.N., Gandhi T.K., Seger A.C. (2005). Patient-reported medication symptoms in primary care. Arch Intern Med.

[66] Derkyi-Kwarteng A.N.C., Agyepong I.A., Enyimayew N., Gilson L. (2021). A narrative synthesis review of out-of-pocket payments for health services under insurance regimes: a policy implementation gap hindering universal health coverage in sub-Saharan Africa. Int J Health Policy Manag.

[67] Yenet A., Nibret G., Tegegne B.A. (2023). Challenges to the availability and affordability of essential medicines in African countries: a scoping review. Clin Outcomes Res.

[68] Shahin W., Kennedy G.A., Stupans I. (2019). The impact of personal and cultural beliefs on medication adherence of patients with chronic illnesses: a systematic review. Patient Prefer adherence.

[69] Fang W., Zhang W.-B., Woods D. (2026). Urban–rural disparities in geographic healthcare accessibility: a comparative study of Nigeria and Zambia: W. Fang et al.. J Urban Health.

[70] Aliaga L., Aliaga A., Smilkov K., Naumovska Z., Gjorgieva Ackova D. (2025). Improving medication adherence: A review of assessment methods and interventions. Int J Innov Res Sci Stud.

[71] Ernawati I. (2022). The effect of the number of drugs received on the level of adherence to drug consumption (Morisky, Green, Levine Adherence Scale Questionnaire) of hypertensive patients. Effect Number Drugs Received Level Adherence Drug Consumption (Morisky Green Levine Adherence Scale Questionnaire) Hypertensive Patients.

[72] Grymonpre R.E., Didur C.D., Montgomery P.R., Sitar D.S. (1998). Pill count, self-report, and pharmacy claims data to measure medication adherence in the elderly. Ann Pharmacother.

[73] Elseviers M., Vrijens B. (2024). Assessment of medication adherence in field research. Drug Util Res Methods Appl.

[74] Beyene K.A., Sheridan J., Aspden T. (2014). Prescription medication sharing: a systematic review of the literature. Am J Public Health.

