# Supplementary material for: Medication non-adherence and its associated factors among heart failure patients in Africa: A systematic review and meta-analysis
Source: Glob Health Res Policy. 2026 Jun 26;11(1):175–84. doi: 10.1016/j.ghrp.2026.06.007 (PMC13722073; doi:10.1016/j.ghrp.2026.06.007)
Supplement: Supplementary file 1 [file mmc1.docx]

Table S2a: JBI Critical Appraisal Summary – Analytical Cross‑Sectional Studies on Medication Non‑Adherence Among Heart Failure Patients in Africa (14 Studies).

| Study (Author, Year) | Q1 | Q2 | Q3 | Q4 | Q5 | Q6 | Q7 | Q8 | Total Yes |
| --- | --- | --- | --- | --- | --- | --- | --- | --- | --- |
| 1. Tarekegn et al. 2025 | Y | Y | Y | Y | U | Y | Y | Y | 7 |
| 2. Azizew et al. 2025 | Y | Y | Y | NA | Y | Y | Y | Y | 7 |
| 3. Dagnew et al. 2024 | Y | Y | Y | Y | U | Y | Y | Y | 7 |
| 4. Tsige et al. 2024 | Y | Y | Y | Y | U | Y | Y | Y | 7 |
| 5. Hussein et al. 2024 | Y | Y | Y | Y | U | Y | Y | Y | 7 |
| 6. Seid et al. 2023 | Y | Y | Y | Y | N | Y | Y | Y | 7 |
| 7. Damte et al. 2022 | Y | Y | Y | Y | Y | Y | Y | Y | 8 |
| 8. Seid et al. 2020 | Y | Y | Y | Y | Y | Y | Y | Y | 8 |
| 9. Fetensa et al. 2019 | Y | Y | Y | Y | Y | Y | Y | U | 7 |
| 10. Ruf et al. 2010 | Y | Y | Y | U | U | Y | Y | Y | 6 |
| 11. Awad et al. 2017 | Y | Y | Y | U | U | Y | Y | U | 5 |
| 12. Ale et al. 2021 | Y | Y | Y | Y | U | Y | Y | U | 6 |
| 13. Shoulkamy et al. 2018 | Y | Y | Y | Y | U | Y | Y | U | 6 |
| 14. Ragbaoui et al. 2017 | Y | Y | Y | U | U | Y | Y | U | 5 |

Tool: Joanna Briggs Institute (JBI) Checklist for Analytical Cross‑Sectional Studies (8 items)
Scoring: Y = Yes, N = No, U = Unclear, NA = Not applicable

**Cross-sectional Checklist Questions**

**Q1:** Inclusion criteria clearly defined?

**Q2:** Subjects and setting described in detail?

**Q3:** Exposure measured validly and reliably?

**Q4:** Objective, standard criteria used for measurement of condition?

**Q5:** Confounding factors identified?

**Q6:** Strategies to deal with confounding factors stated?

**Q7:** Outcomes measured validly and reliably?

**Q8:** Appropriate statistical analysis used?

Table S2b: JBI Critical Appraisal Summary – Cohort Studies on Medication Non‑Adherence Among Heart Failure Patients in Africa (3 Studies).

| Study (Author, Year) | Q1 | Q2 | Q3 | Q4 | Q5 | Q6 | Q7 | Q8 | Q9 | Q10 | Q11 | Total Yes |
| --- | --- | --- | --- | --- | --- | --- | --- | --- | --- | --- | --- | --- |
| 1. Pallangyo et al. 2020 | Y | Y | Y | Y | Y | U | Y | Y | N | U | Y | 8 |
| 2. Yayehd et al. 2012 | Y | Y | NA | Y | Y | U | Y | Y | N | U | Y | 7 |
| 3. N'Cho-Mottoh et al. 2015 | Y | Y | Y | Y | Y | U | Y | Y | N | U | Y | 8 |

Tool: Joanna Briggs Institute (JBI) Checklist for Cohort Studies (11 items)
Scoring: Y = Yes, N = No, U = Unclear, NA = Not applicable

**Cohort Studies Checklist Questions**

**Q1:** Two groups similar and recruited from same population?

**Q2:** Exposures measured similarly for both groups?

**Q3:** Exposure measured validly and reliably?

**Q4:** Confounding factors identified?

**Q5:** Strategies to deal with confounding factors stated?

**Q6:** Groups free of outcome at start of study?

**Q7:** Outcomes measured validly and reliably?

**Q8:** Follow-up time reported and sufficient?

**Q9:** Follow-up complete, and reasons for loss to follow-up described?

**Q10:** Strategies to address incomplete follow-up utilized?

**Q11:** Appropriate statistical analysis used?
